# A Rising Star in Pancreatic Diseases: Pancreatic Stellate Cells

**DOI:** 10.3389/fphys.2018.00754

**Published:** 2018-06-18

**Authors:** Ran Xue, Kai Jia, Jianxin Wang, Lixin Yang, Yanbin Wang, Lingyun Gao, Jianyu Hao

**Affiliations:** Department of Gastroenterology, Beijing Chao-Yang Hospital, Capital Medical University, Beijing, China

**Keywords:** pancreatic stellate cell, stem/progenitor cell characteristics, exosomes, cellular senescence, epithelial mesenchymal transformation, energy metabolism, direct mechanical reprogramming

## Abstract

Pancreatic stellate cell (PSC) is a type of pluripotent cell located between pancreatic lobules and the surrounding area of acinars. When activated, PSC can be transformed into myofibroblast-like cell. A number of evidences suggest that activated PSC is the main source of the accumulation of extracellular matrix (ECM) protein under the pathological conditions, which lead to pancreatic fibrosis in chronic pancreatitis and pancreatic cancer. Recent studies have found that PSC also plays an important role in the endocrine cell function, islet fibrosis and diabetes. In order to provide new strategies for the treatment of pancreatic diseases, this paper systematically summarizes the recent researches about the biological behaviors of PSC, including its stem/progenitor cell characteristics, secreted exosomes, cellular senescence, epithelial mesenchymal transformation (EMT), energy metabolism and direct mechanical reprogramming.

## Introduction

**FIGURE 1:**
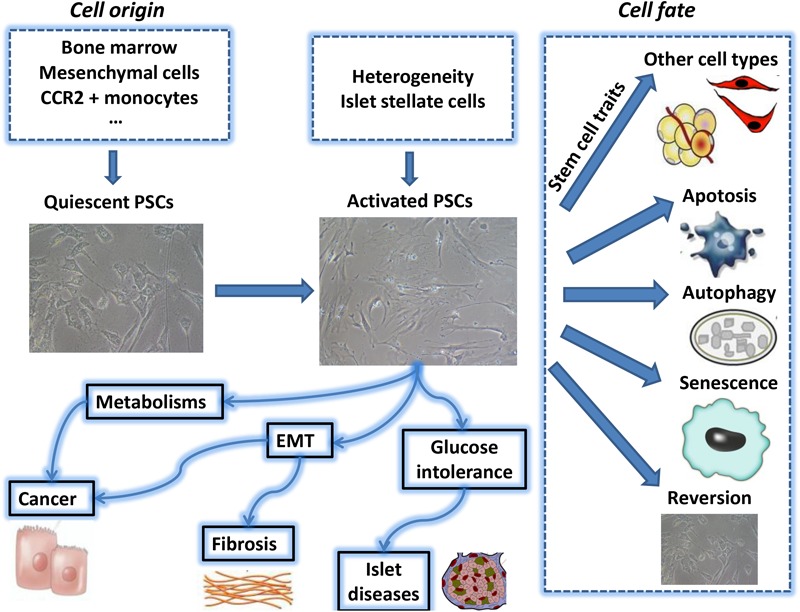
The above summarizes the major topics of biological behavior of pancreatic stellate cell which are covered during this review period. PSC, pancreatic stellate cell; EMT, epithelial-mesenchymal transition.

Pancreatic stellate cells (PSCs) are exocrine functional myofibroblasts which found in the pancreas. PSC is regulated by autocrine and paracrine stimulation, and has many similar biological characteristics of HSC. Previous studies on HSC biological mechanisms can contribute to the further study of PSC biological behavior. In the study of CP and pancreatic cancer-related fibrosis, the roles of sustained activation of PSCs cause more and more attention. In-depth understanding of the biological characteristics of PSCs will lay the foundation for further exploration of the PSC function in various pancreatic diseases and provide a novel insight for PSC targeted therapy. A summary of the key areas discussed in this review can be found in **Figure 1**.

## The Naming of PSCs

In 1876, Germany researcher Carl von Kupffer first described the stellate cells (Sternzellen) and successively found similar cells in the kidney (Liu, 2006) and lung (Keane et al., 2005). Then in 1982, the Japanese scholars Watari et al. (1982) observed HSC-similar cells, which have vitamin A stored function in the mouse pancreatic tissue using autofluorescence and electron microscopy technology. But only until 1998, two groundbreaking reports described the separation, culture, and characteristic expression of this type cell (Apte et al., 1998; Bachem et al., 1998), which was eventually named PSC.

## The Type of PSCs

**FIGURE 2:**
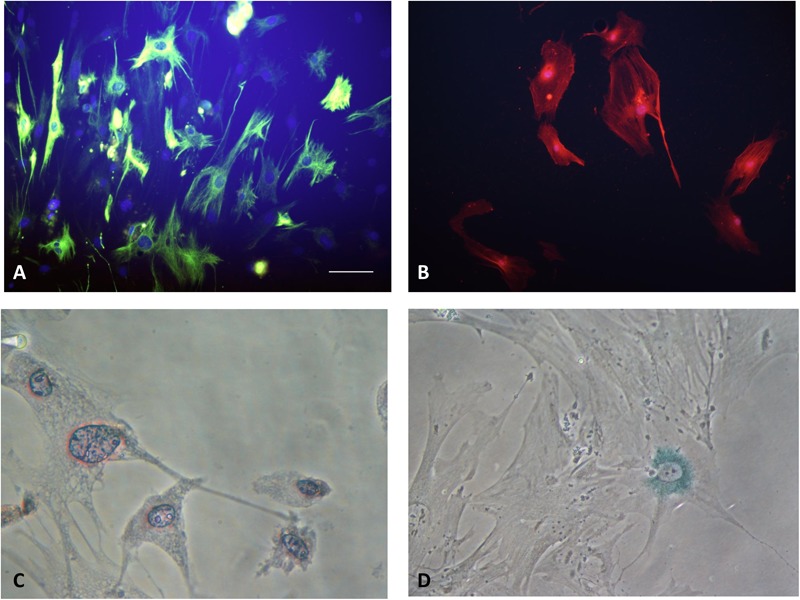
**(A)** Quiescent PSCs stain positively for desmin by IF. (Hoechst 33258 staining, and Alexa Fluor 488 staining for desmin, original magnification, ×200). **(B)** Activated PSCs stain positively for α-SMA by IF (Hoechst 33258 staining, and Alexa Fluor 594 staining for α-SMA, original magnification, ×200). **(C)** Quiescent PSCs have an angular appearance, contained lipid droplets by oil red O staining. (Original magnification, ×400). **(D)** Senescence is shown in PSCs with cytoplasmic blue staining of SA-β-gal. (Immunocytochemistry; original magnification, ×400) (Xue et al., 2017b).

Pancreatic stellate cells can be divided into two phenotypes: quiescent and activated. In normal physiological state, PSCs stay in quiescent phenotype. Quiescent PSCs express nestin, vimentin, GFAP and desmin (**Figure 2A**). Quiescent PSCs also contain retinoids, predominantly as retinyl palmitate cytosolic droplets, which can be used to distinguish PSCs from normal fibroblasts (**Figure 2C**). A recent study identified the markers enabling the identification of quiescent PSCs in normal human paraffin embedded and formalin-fixed pancreatic tissue, which suggested that cytoglobin and adipophilin are bio-markers of quiescent PSCs in the normal human pancreas (Nielsen et al., 2017). It also indicated that the expression of PSCs markers may vary across different species.

When stimulated by various pathogenic factors, the quiescent PSC is activated, transformed into activated myofibroblast-like cell. The process of PSC phenotype transition is correlated to functional and morphological changes. It’s widely accepted that PSCs are loss of retinoid droplets from the cytosol and increase expression of α-SMA (**Figure 2B**). Activated PSCs have an increase in production of laminin, fibronectin, collagen type I and III, as well as actively migrate and proliferate (Apte et al., 2013a; Xue et al., 2017b). The characteristics of different type PSCs were summarized in the **Table 1**.

**Table 1:** The characteristics of different type PSCs.

	Activated PSCs	Quiescent PSCs
α-SMA	Yes	No
Vitamin A lipid droplets	No	Yes
Vimentin	Less	More
Glial fibrillary acidic protein (GFAP)	Less	More
Desmin	Less	More
Migration	Enhanced	Limited
Proliferation	Enhanced	Limited
Extracellular matrix production	Enhanced	Limited
Matrix metalloproteinases (MMPs) and tissue inhibitors of matrix metalloproteinases; (TIMPs)	Loss of balance between MMPs and TIMPs	Secrete MMPs and TIMPs
Cytokines, chemokines and growth factors	Secrete various cytokines, chemokines and growth factors (PDGF, TGFβ, CTGF, IL1,IL6, IL15)	No or limited secretion of cytokines, chemokines and growth factors
Functions	Contribute to the fibrosis and hypoxic tumor microenvironment	Involved in maintenance of pancreatic tissue architecture
	Involved in angiogenesis and epithelial-mesenchymal transition	Function as an immune, progenitor and intermediary cell

## The Origin of PSCs

The results of transcriptomics and proteomics analysis showed that there were significant similarities between HSCs and PSCs, despite the presence of organ specificity (Paulo et al., 2013). At the same time, the two are different from skin fibroblasts. As for the different characteristics between PSCs and HSCs, a recent studies showed that PSCs had a very small possession of vitamin A using mass spectrometry and a low expression of lecithin retinol acyltransferase. Meanwhile, the microstructure of PSCs was entirely different from that of HSCs by flow cytometry and electron microscopy (Yamamoto et al., 2017). Because stellate cells express both mesenchymal markers and neurotrophic factors, the lineages of stellate cells are always a hot topic in the field. Cell lineage tracing studies have confirmed that HSC is derived from mesenchymal cells and have evolved from a mesodermal origin (Cassiman et al., 2006; Asahina et al., 2011). Similar studies for PSCs are awaited.

In the context of CP and PDAC, a proportion of PSCs are thought to originate in the bone marrow (Scarlett et al., 2011). The speculation that bone marrow is another potential source of PSCs was further supported by a recent study involving dibutylin chloride induced CP (Sparmann et al., 2010). A recent study reported that CCR2^+^ monocytes can migrate to the pancreas through the MCP-1/CCR2 pathway and differentiate into PSCs (Ino et al., 2014). In some degree, the heterogeneity of PSCs can be explained by the fact that some cells are derived from hematopoietic cells and other from mesodermal cells. The in-depth exploration for the origin of PSCs could help us to discover the essence of these kind of cells. According to the origin of PSCs, we can speculate function that PSCs may have and its clinical significance.

## The Functional Heterogeneity of PSCs

Recently, research into cancer biology has focused on the concept of CSCs. CSCs have the ability to initiate and sustain tumor formation (O’Brien-Ball and Biddle, 2017; Xue et al., 2017a). CSCs were isolated on the basis of their expression of cell surface markers such as CD44, CD24, and CD133.

Based on the origin of PSCs and the concept of CSCs, functional heterogeneity of PSCs has been proposed recently. PSCs comprise several different cell subpopulations on the basis of the different cell surface markers. Different cell subpopulations hold the diverse function in pancreatitis and in cancer as an eventual promoter/preventer of cancer progression. In other words, better highlight and summarize the functional heterogeneity of PSCs that were shown to favor suppress cancer development and metastasis.

It has been reported that compared with CD10-PSCs, CD10^+^ PSCs had the capacity to promote the invasiveness of cancer cells and were responsible for subcutaneous tumor growth (Ikenaga et al., 2010). S100A4, another new bio-marker for activated PSCs, has been reported in murine pancreatic cancer-related fibroblasts (Farrow et al., 2009; Zechner et al., 2014). Birtolo et al. (2017) discovered that cadherin-11 was also up-regulated in activated PSCs. And its mediated cell adhesion and TGF-β signaling participated in cancer cell migration involved in the spread and metastasis of pancreatic cancer. CD271+ PSCs existed near tumor and played a part in the resistance to carcinogenesis and progression of PDAC (Fujiwara et al., 2012). In mouse xenograft experiments, the high levels of palladin expression in CAFs can enhance the rapid growth and metastasis of tumor cells by promoting the invadopodia formation in pancreatic cancer-related fibroblasts (Goicoechea et al., 2014). A recent study reported that the functional heterogeneity of PSCs about HGF-mediated tumor-stroma interactions suggested that inhibition of the HGF pathway in PDAC have disparate effects in diverse subsets of patients (Tjomsland et al., 2017). Immunohistochemical analysis confirmed that ATP-gated receptor P2X7 (P2X7R) expressed in tumor and PSCs. AZ10606120 is an P2X7 antagonist. PSCs number/activity, as well as collagen deposition was reduced in AZ10606120-treated tumors (Giannuzzo et al., 2016). Kindlins are essential regulators of integrin signaling and integrin-mediated cell adhesion to the ECM (Larjava et al., 2008; Montanez et al., 2008). Kindlin-2 in PSCs can promote the progression of PDAC. The expression of stromal kindling-2 was related to shorter recurrence-free survival time after R0 resection (Yoshida et al., 2017). A recent study implicated PAK1 can regulate PSC activation, apoptosis and proliferation. Targeting stromal PAK1 may increase survival and therapeutic response of patients with PDAC (Yeo et al., 2017). Öhlund et al. (2017) revealed another distinct subpopulation of PSCs, located more distantly from neoplastic cells, which lacked elevated α-SMA expression and instead secreted IL6 and additional inflammatory mediators in 2D and 3D co-cultures system, which is the first to characterize the intratumoral CAF heterogeneity in PDAC. A summarizing PSC biomarkers associated with pancreatic cancer promotion/invasiveness versus repression were shown in **Table 2**. And it also represented the potential anticipated molecules for the future treatment of pancreatic diseases.

**Table 2:** A summarizing PSCs biomarkers associated with pancreatic cancer promotion/invasiveness and suppression.

Year	PSCs biomarkers	Function
2009	S100A4	S100A4 is key component of the pancreatic tumor stroma.
2010	CD10	CD10^+^ PSCs enhance the progression of pancreatic cancer.
2012	CD271	CD271^+^ subpopulation of PSCs correlates with prognosis of pancreatic cancer and is regulated by interaction with cancer cells.
2014	Palladin	Palladin promotes invasion of pancreatic cancer cells by enhancing invadopodia formation in cancer-associated fibroblasts.
2016	P2X7R	P2X7R expressed in tumor and PSCs, which is related to PSCs number/activity, as well as collagen deposition.
2017	HGF	HGF show the functional heterogeneity in tumor-derived human PSCs, which implicated the mitogenic signaling and migration in pancreatic cancer.
2017	Cadherin-11	Cadherin-11 is a cell surface marker up-regulated in activated PSCs and is involved in pancreatic cancer migration.
2017	Kindlin-2	Kindlin-2 in PSCs promotes the progression of pancreatic cancer.
2017	PAK1	Inhibition of PAK1 suppresses PSCs activation and increases survival of mice with pancreatic cancer.
2017	iCAFs and myCAFs (αSMA and IL-6)	CAFs coexist as two mutually exclusive and reversible subtypes: iCAFs and myCAFs, which have different location in pancreas and hold different function.

To sum up, PSCs have functional heterogeneity, which means that PSCs comprise several other cell subpopulations which can individually or synergistically influence the progression of PDAC. The exploration for the bio-markers of PSCs is essential for the identification of heterogeneity of PSCs, which is helpful in identifying the cellular origin and reflects the malignant essence of PDAC.

Actually, mesenchymal cells and myofibroblasts isolated from various tissues also exhibit different phenotypes (Polisetty et al., 2008; Strakova et al., 2008). Strobel et al. (2016) cultured primary PSC derived from normal pancreas, CP and PDAC in a low-serum environment. Comparative analysis showed that PSCs isolated from different contexts maintain different phenotype, which is also the reflection of the stromal activities in original their tissue. In addition, it is reported that the composition of ECM also significantly affects PSC gene expression patterns (Apte et al., 2013b). The current opinion holds that PSC-stroma-cancer cell interaction is dynamic and stage-dependent, with a protective effect on the earliest stage and harmful effect in later stages (Bynigeri et al., 2017). The biophysical aspects of PSC/ECM interactions in stromal reprogramming are also opening new avenues for management of PSC related diseases. Meanwhile, detailed characterization of PSCs in PDAC can be conducive to clarify the mechanism underlying the interactions between tumor cells and stromal cells, which would provide novel targets for stroma-directed treatments.

## Islet Stellate Cells (ISCs)

Zha et al. (2014a) used collagens to digest the pancreas islet of rat to obtain isolated ISCs. These cells expressed PSC-related bio-markers. At the same time, these cells had less lipid droplets than typical PSCs, and were easier to be activated and turned into α-SMA+ cells. But their proliferation and migration rates were significantly lower than typical PSCs. Recently, Zha et al. (2016) used standard explants techniques to isolate the corresponding ISCs in human pancreatic tissue, which were also different from the typical PSCs. Human ISCs express α-SMA, desmin, vimentin and GFAP, as well as collagen I, collagen III and fibronectin. Both human PSCs and ISCs are capable of *trans*-differentiating into adipocytes and osteoblast-like cells *in vitro*.

Islet stellate cells can act as subgroups of PSCs and interact with pancreatic β-cells, included enhancing β-cell apoptosis, inhibiting proliferation, and decreasing β-cell function, which are important in islet fibrosis and islet cell dysfunction (Li et al., 2016; Zha et al., 2016).

As for the relationship between PSCs and islets, it is currently accepted that PSCs not only promote islet fibrosis, but also have relation to glucose intolerance in some diseases (Zha et al., 2014b; Zang et al., 2015; Lee et al., 2017). In addition, PSCs can regulate the immune response and improve the survival rate of islet transplanted through stimulating graft vascular regeneration (Vonlaufen et al., 2008; Guan et al., 2015; Hsieh et al., 2015). Zang et al. (2015) found that the activated PSCs have a direct impact on pancreatic islet cell viability and proliferation rate. Meanwhile, PSCs can induce β-cell failure *in vitro* and *in vivo*. We consider that the type of PSCs associated with glucose intolerance may belong to ISCs. Activated PSCs were conducive to impaired islet endocrine function seen in islet fibrosis and in exocrine pancreatitis, which indicated that PSCs involved in the pathogenesis of type 2 diabetes. It opens up for novel therapy insight for type 2 diabetes based on regulation of the activated process of PSCs.

## Stem/Progenitor Cell Characteristics of PSCs

Many studies suggested that stellate cells are related to cells of the hematopoietic system and share some characteristics with hematopoietic stem/ progenitor cell. This leads to the interesting question: Are PSCs undifferentiated cells?

The rodent’s pancreas has regenerative capacity (Fitzgerald and Alvizouri, 1952). It has been found that certain PSCs isolated from pancreatic tissue express a variety of stem/progenitor cell markers such as nestin, CD133, CD34, Aldh, p75NTR, Bcrp1 and factors involved in developmental processes, such as GDF3 and PITX2. In addition, as the signal pathways for maintaining the development of stem cells, β-catenin-dependent Wnt, Hedgehog, Jak/Stat, Activin/Tgf/Nodal and Notch signals are involved in functional regulation of PSCs (Niwa, 2001; Lengerke et al., 2008; Kordes et al., 2009; Senoo et al., 2017). PSC clones preserved the expression of stem/progenitor cell and stellate cell markers, as well as maintained their differentiation potential, which suggested PSCs have self-renewal potential. All these findings demonstrate that PSCs have stem/progenitor cell characteristics and can contribute positively to the regeneration of injured tissue by differentiation across organs boundaries. At the same time, PSCs can directly promote liver regeneration by differentiating into hepatocytes and bile ducts (Kordes et al., 2012). The function of PSCs have not only confined to interstitial support, but can be used as mesenchymal stem cells, through differentiating into epithelial cell lineages for assisting in the regeneration of damaged tissue (Claus et al., 2014).

An important study from Mato et al. (2009) identified a population of PSCs that express the ABCG2+ transporter and can transdifferentiate into insulin-producing cells; exhibit the properties of stem/progenitor cells. Co-culture of PSCs and pancreatic cancer cells can enhance the ability of globular formation of tumor cells and induce the expression of nestin, LIN28 and ABCG2. Furthermore, co-injection of PSCs can enhance tumorigenicity in tumor cells, which means PSCs can enhance the tumor stem cell-like phenotype of pancreatic cancer cells (Hamada et al., 2012). Meanwhile, activated PSCs are indeed playing a stimulatory role in replication of mature pancreatic acinar cells and islet cells through the activity of secreted collagen *in vivo* (Shigenori et al., 2016). Kruse et al. (2011) pointed to a potential for adipocyte differentiation in human PSCs. Peroxisome proliferator-activated receptor gamma ligands play an important role in the differentiation potential of PSCs. In a word, stem/progenitor cell characteristics of PSCs means that PSCs are involved in the pancreatic regeneration. And it is also indicated that PSCs can be used in the area of cell reprogramming. PSC may become a type of tool cell for the exploration of novel regulation mechanism of somatic cell reprogramming.

Due to the undifferentiated state and the capability of expressing peculiar stem genes, PSCs play an important role during pancreatic regeneration. On the contrary, a number of other body cell types can also express progenitor/stem cell biomarkers, therefore, further transplantation studies with extremely enriched stellate cell preparations are still required for a final conclusion.

Another trait of stem cells that should be tested for stellate cells is transplantability. The argument about the classification of freshly isolated PSCs is still on the table, because molecular markers of progenitor and stem cells are both present. Further studies are still required.

## The Exosomes Secreted by PSCs

It has been more and more recognized that extracellular vesicles (EVs) involving exosomes are significant mediators of cell-to-cell communications (Yáñez-Mó et al., 2015). Exosomes are membrane-enclosed nanovesicles containing diverse host cell-derived bioactive molecules including lipids, proteins, as well as microRNAs (miRNAs).

In pancreatic cancer, the exosome-mediated communications contribute to optimize conditions for the growth and metastasis of cancer. Takikawa et al. (2017) reported that PSC-derived exosomes, containing a variety of miRNAs, were used by pancreatic cancer cells, resulting in the stimulation of migration, proliferation, and chemokine gene expression in tumor. PSC-derived annexin 6A-positive (ANXA6+) EVs containing the annexin A6/LDL receptor-related protein 1/thrombospondin1 (ANXA6/LRP1/TSP1) complex promotes pancreatic cancer aggressiveness following uptake via tumor cells, and ANXA6 depletion by infection of shANXA6 in CAFs impaired tumor metastasis (Leca et al., 2016). Moreover, PSC-derived exosomes can rescue the proliferation of nutrient-deprived MiaPaCa-2 and BxPC3 cells in a KRAS-independent manner through supplying them with metabolites (Zhao et al., 2016).

As for the effect of exosomes derived from PSCs exposed to chemotherapy, Richards et al. (2017). showed that exosomes released from gemcitabine-treated CAFs increased the proliferation and survival of recipient epithelial cancer due to the increased level of Snail and its target, miR-146a, in recipient cells. Furthermore, supression of exosome secretion from CAFs decreased pancreatic cancer cell proliferation and survival.

In CP, Charrier et al. (2014) found that microRNA-21 and connective tissue growth factor (CCN2) are components of a positive feedback loop in PSCs and are exported in PSC-derived exosomes.

Transfer via exosomes seems to be multidirectional. Further research is urgently needed to clarify the interactions between parenchyma cell and mesenchymal cells such as PSCs, as well as the factors including exosomes, that mediate intercellular communication to promote and sustain malignant transformation and metastasis.

## PSCs and Cell Senescence

Cell senescence can be defined as an irreversible model of cell cycle arrest. It can limit the proliferation potential of precancerous cells, which is an important barrier against tumorigenesis (Campisi and d’Adda di Fagagna, 2007). Cell senescence is associated with replication depletion caused by telomere shortness and can be triggered by different forms of cell damage or stress. Once the senescence process is activated, the cells will stop dividing and undergo characteristic metabolic and morphological changes (**Figure 2D**). Many senescent cells, including HSCs, exhibit senescence-associated secretary phenotypes (SASP). They can alter the genes that secrete proteins in the tissue microenvironment through coding over-expression (Campisi and d’Adda di Fagagna, 2007; Krizhanovsky et al., 2008).

Fitzner et al. (2012) found that cultured PSCs in an exposed environment of stress factors (doxorubicin, H_2_O_2_ and staurosporine) for a long-term, can induce cell senescence. Senescent PSCs highly express CDKN1A/p21, mdm2 and IL-6, and lowly express α-SMA. Meanwhile, CDKN1A/p21 plays a direct role in the initiation/progression of PSCs. Inhibition of cell proliferation alone is not enough to induce PSCs senescence, and PSCs senescence is regulated by multiple independent major signaling pathways.

In CP, the number of senescent cells is significantly associated with the severity of inflammation and fibrosis. Both the fibrotic region and senescence-associated β-Galactosidase (SAβ-Gal) staining positive region overlap with the dense infiltrating region of the immune cells. In addition, a close physical proximity of activated PSCs and immune cells was observed. These results suggest that inflammation, cellular senescence and PSCs activation are timely coupled processes which occur in the same microenvironment of inflamed pancreas. Moreover, lymphocytes have a dual-specific role in pancreatic fibrogenesis, triggering the beginning of wound healing by activated PSCs, and its completion by killed senescent PSCs (Fitzner et al., 2012). Further studies have confirmed that senescence is significantly associated with activation of PSCs, which may be due to the potentially critical role of SASP in the matrix environment. In the tumor microenvironment, SASP-related secretory factors promote the activation of surrounding stoma cells and induce tumorigenesis (Moir et al., 2014; Porciuncula et al., 2016). Cell senescence determines the fate of activated PSCs.

Some studies have shown that Cdkn1a plays a direct role in the process of rat PSC senescence (Fitzner et al., 2013). Cdkn1a independent pathways may help to maintain a typical PSC senescence gene expression profile. The knockdown of Cdkn1a significantly reduced the inhibitory effect of doxorubicin on PSC growth and SA β-Gal positive cells. Over-expression of Cdkn1a enhances the anti-proliferative effect and induced senescence effect of doxorubicin on PSCs. In the primary PSCs, the treatment of doxorubicin can increase the expression of IL-6 and MMP-9, and reduce the expression of α-SMA, p53, Cdk1 and Rad54. In short, cell senescence could be a mechanism for regulating PSCs function.

## PSCs and Epithelial-Mesenchymal Transition (EMT)

Epithelial mesenchymal transformation is a biological phenomenon that epithelial cells lose epithelial properties to obtain stromal cell phenotypes. EMT is commonly found in embryonic development, tissue regeneration, organ fibrosis and tumor formation (Tzanakakis et al., 2017). In the process of EMT, the expression of epithelial markers, such as E-cadherin and cytokeratins, were down-regulated; the expression of interstitial markers, such as vimentin, fibronectin and N-cadherin, were up-regulated. The down-regulation of E-cadherin protein was the most important molecular event in EMT (Kishi et al., 2014). Transcription factors that induce EMT mainly include Snail, Slug, STAT3 and Twist (Wendt et al., 2014; Dai et al., 2017). In addition, the changes of cell morphology and enhancement of migration capacity are also important features for EMT. ECM plays an essential role in the occurrence of EMT. EMT is the initial stage of pancreatic tissue fibrosis, and activated PSC is the core of pancreatic fibrosis.

Tian et al. (2016) analyzed the phenotype of PSCs in rat pancreas and found that the migration and motility of activated PSC cells enhanced along with the change of EMT-related gene expression, indicating that the process of activation in PSCs occur the changes like EMT.

It has been reported that miR-200a can reverse EMT by inhibiting TGF-β1-induced PSCs activation and ECM deposition through regulating PI3/Akt and PTEN/Akt/mTOR signal pathway. Plus, miR-429 can inhibit of ECM synthesis to reverse EMT by TGF-β1-mediated TGF-β/Smad signaling (Sivadas and Kannan, 2014; Xu et al., 2017).

Kikuta et al. (2010) determined that PSCs co-cultured pancreatic cancer cells with showed fibroblast-like appearance, loose cell contact and dispersion. The expression of epithelial markers in PSCs decreased, along with the expression of interstitial markers increased and migration ability increased, which confirmed that PSCs can promote the EMT process of pancreatic cancer cells. EMT can be a new mechanism to improve the attack ability of pancreatic cancer cells through the PSCs. The latest 3D pancreatic organ model studies have shown that co-culture with tumor-associated PSCs (TPSCs) could induce changes of EMT bio-markers in pancreatic cancer cells (Karnevi et al., 2016). High glucose conditions can further enhance the changes of EMT markers and cancer cell invasion.

A recent study reported that paracrine IL-6 signaling regulates the effects of PSCs on EMT via Stat3/Nrf2 pathway in PDAC (Wu et al., 2017). Targeting Stat3/Nrf2 pathway activated by PSC-secreted IL-6 could provide a novel option to improve the prognosis of PDAC. Retinoic acid can inhibit pancreatic cancer cell EMT and migration by the down-regulation of IL-6 in CAFs (Guan et al., 2014).

Progression of PDAC is promoted by desmoplasia induced by PSCs. EMT plays an important role in this progression. The characteristic of EMT is similar to CSCs hypothesis. PSC can enhance the CSC phenotype, as well as the radioresistance of pancreatic cancer cells (Al-Assar et al., 2014). The in-depth study about the relationship between PSCs and EMT could give us more ideas from the PSC-related diseases, especially in the cell invasion and migration.

## PSCs and Tumor Cell Energy Metabolism

Pancreatic ductal adenocarcinoma is a highly virulent digestive system tumor characterized by significant fibrotic matrix responses and energy metabolism disorders (Sousa and Kimmelman, 2014; Whatcott et al., 2015). In the process of tumor progression, the Warburg effect, autophagy and glutamine addiction are the most significant metabolic mechanisms of tumor cells. At the same time, energy metabolism reprogramming is also a vital feature of tumor microenvironment (Zhao et al., 2016).

As the main cell type in the pancreatic tumor matrix, PSC is a substantial mediator of connective tissue hyperplasia, and its metabolites affect the metabolism of tumor cells. For the cell growth and metabolism of matrix PSC and epithelial cancer, the two are mutually reinforcing relationship. The metabolism mutual promotions within the tumor may occur in different groups of tumor cells.

The activation of PSC is accompanied by rapid growth and proliferation of cells, as well as the expansion of mitochondria and ER. The resting state PSCs and activated PSCs have significant metabolic changes because of the requirements of biological energy and metabolic synthesis (Nielsen et al., 2017; Xue et al., 2017b). ER stress participates in the phenotypic transformation of PSC cells. The unfolded protein response plays a major role to steady the PSC mitochondrial stress response. At the same time, metabolic stress-induced PSC reprogramming is related to the regulation of adjacent cells in the tumor microenvironment (Su et al., 2016).

Recent studies have shown that PSC-induced changes in PDAC metabolism can promote tumor cell proliferation under nutrient restriction conditions. PSCs can maintain tumor metabolism through autophagic alanine secretion (Sousa et al., 2016). Meanwhile, autophagy is required for activation of PSCs, correlated with pancreatic tumor progression and promotes growth of pancreatic cancers in mice (Endo et al., 2017).

A new concept, “reverse Warburg effect” (RWE), potentially revolutionizes the way we look at tumor cell behavior and the critical role the stroma plays in its progression (Fu et al., 2017). Stromal CAFs, the tumor-supporting features of PSCs, the dominating fibroblastic cell type in the tumor microenvironment of the pancreas, represents a potential therapeutic target. The RWE demonstrates that CAFs, which is similar to the activated PSC form, are the central site of aerobic glycolysis. Hydrogen peroxide is secreted through tumor cells and this causes oxidative stress in neighboring fibroblasts, leading to the activation of CAFs, autophagy, glycolysis and overall catabolism. It has been found that metformin can adjust the cell function of PSCs and reproduce the oxidative stress of cancer-associated fibroblasts by reprogramming PSCs (Nair et al., 2014; Qu and Yang, 2015). Metabolic reprogramming of stromal fibroblasts promotes inflammation and tumorigenesis by p62-mTORC1 signaling (Valencia et al., 2014). Above all, PSCs play an important role in tumor cell energy metabolism, and the mechanisms are related to antophagy and oxidative stress, which provide a novel insight to treatment of pancreatic cancer.

## PSCs and Direct Mechanical Reprogramming

The tumor environment contributes significantly to cancer cell behavior and tumor progression (Wei and Yang, 2016). Physical factors of the environment also affect the cancer aside from biochemical constituents. More and more evidences suggest that mechanics, such as tissue pressure, tumor (stroma) elasticity, are key players of tumor progression (Levental et al., 2009; Schrader et al., 2011; Quinlan and Billiar, 2012; Ivey et al., 2016). Underlying mechanobiological mechanisms include the MS ion channels of cancer-associated cells, the regulation of focal adhesion molecules, and cytoskeletal modifications.

In fact, PDAC is one of the most stroma-rich and fibrotic malignancies intuitively contributes to the insight that ECM mechanics play a core role in the development of fibrosis and PDAC progression. PDAC is characterized by the formation of a dense fibrotic stroma (desmoplasia), which formed by activated PSCs. Desmoplasia leads to high tissue pressure, which in turn activates PSCs, thereby perpetuating ECM deposition (Waghray et al., 2013; Zhan et al., 2017).

Fels et al. (2016) pointed that mechanistic insight how pressure, a significant factor of the tumor environment, leads to the activation of PSC. TRPC1-mediated activation may be a potent target to disrupt the positive feedback of PSC activation and PDAC progression. Storck et al. (2017) showed that ion channels are essential players in PSC-related physiology and pathophysiology. A recent research demonstrated that matrix stiffness regulates activation and mechanotaxis in PSCs through the use of a physiomimetic system. As a result of changes in ECM stiffness, PSCs acquired the ability to undergo phenotypic transition. Meanwhile, it is observed that the ability of PSCs to durotactically respond to stiffness variations within their local environment (Lachowski et al., 2017). A new study reported that ATRA mechanically reprograms PSCs through reducing the ability of PSCs to produce high traction forces and fit extracellular mechanical cues, to suppress force-mediated ECM remodeling for inhibiting tumor cell invasion in 3D organotypic models (Chronopoulos et al., 2016). Meanwhile, ATRA can modulate mechanical activation of TGF-β by PSCs (Sarper et al., 2016).

Based on the findings above, it is indicated that the mechanical microenvironment is a potential contributor to PDAC progression by induction of PSC activation and pancreatic fibrosis, which means that direct mechanical reprogramming of PSCs is a viable choice in the treatment of PSC-related disease, such as CP and PDAC.

## Conclusion and Expectation

With the role of PSCs in CP and pancreatic cancer more and more clearly, many studies are paying attention to targeted activation of PSC treatment. This treatment strategy will be expected to reduce the fibrosis of CP, thereby delaying the development of exocrine and endocrine dysfunction. In blocking matrix reaction, PSC can interact with pancreatic cancer cell, thereby inhibiting tumor progression and improving disease prognosis. At the same time, substantial evidence suggests that PSCs also plays an important role in the endocrine cell function, islet fibrosis and diabetes. In order to better explore the mechanisms of pancreatic diseases, the PSC immortalization problem is a positive direction for all of us to explore. Meanwhile, more and more new technologies have been applied in the studies of PSCs, such as 3D PDAC stroma rich spheroid model (Lee et al., 2018). These new technologies will provide an improved knowledge of PDAC biology and has the potential to offer an insight into pathways that may be therapeutically targeted to inhibit PSC activation, thereby interrupting PSC-PDAC cell interactions and suppressing the development of PDAC. This direction can provide great possibilities of major breakthroughs for the pancreas disease treatment.

## Author Contributions

RX drafted the manuscript. YW and LY critically revised the manuscript. JH designed the manuscript. LG and KJ conceived the topic of the review article. KJ and JW collected and reviewed articles.

## Conflict of Interest Statement

The authors declare that the research was conducted in the absence of any commercial or financial relationships that could be construed as a potential conflict of interest.

## Abbreviations

Aldh: aldehyde dehydrogenase
α-SMA: α-smooth muscle actin
Bcrp1: breast cancer resistance protein1
CAF: cancer associated fibroblast cell
CP: chronic pancreatitis
CSCs: cancer stem cells
ECM: extracellular matrix
EMT: epithelial mesenchymal transformation
ER: endoplasmic reticulum
GDF3: growth differentiation factor 3
GFAP: glial fibrillary acidic protein
HSC: hepatic stellate cell
ISCs: islet stellate cells
MS: mechanosensitive
p75NTR: p75 neurotrophin receptor
PAK1: p21-activated kinase 1
PDAC: pancreatic ductal adenocarcinoma
PITX2: paired like homeodomain 2
PSC: pancreatic stellate cell
S100A4: S100 calcium-binding protein A4
